# The Sedative Effect of Non-Alcoholic Beer in Healthy Female Nurses

**DOI:** 10.1371/journal.pone.0037290

**Published:** 2012-07-18

**Authors:** Lourdes Franco, Cristina Sánchez, Rafael Bravo, Ana B. Rodríguez, Carmen Barriga, Eulalia Romero, Javier Cubero

**Affiliations:** 1 Neuroimmunophysiology and Chrononutrition Research Group, Department of Physiology, Faculty of Sciences, University of Extremadura, Badajoz, Spain; 2 SES, Hospital Universitario Infanta Cristina, Badajoz, Spain; 3 Laboratory of Health Education, Science Education Area, University of Extremadura, Badajoz, Spain; Université Pierre et Marie Curie, France

## Abstract

**Introduction:**

The hop (*Humulus lupulus* L.), a component of beer, is a sedative plant whose pharmacological activity is principally due to its bitter resins, in particular to the α-acid degradation product 2-methyl-3-buten-2-ol. The mechanism of action of hop resin consists of raising the levels of the neurotransmitter γ-aminobutyric acid (GABA), an inhibitory neurotransmitter acting in the central nervous system (CNS).

**Objectives:**

To analyze the sedative effect of hops as a component of non-alcoholic beer on the sleep/wake rhythm in a work-stressed population.

**Methods:**

The experiment was conducted with healthy female nurses (n = 17) working rotating and/or night shifts. Overnight sleep and chronobiological parameters were assessed by actigraphy (*Actiwatch®)* after moderate ingestion of non-alcoholic beer containing hops (333 ml with *0,0% alcohol*) with supper for 14 days (treatment). Data were obtained in comparison with her own control group without consumption of beer during supper.

**Results:**

Actigraphy results demonstrated improvement of night sleep quality as regards the most important parameters: *Sleep Latency* diminished (p≤0.05) in the Treatment group (12.01±1.19 min) when compared to the Control group (20.50±4.21 min), as also did *Total Activity* (p≤0.05; Treatment group = 5284.78±836.99 activity pulses vs Control = 7258.78±898.89 activity pulses). In addition, anxiety as indexed by the State-Trait Anxiety Inventory (STAI) decreased in the Treatment group (*State Anxiety* 18.09±3.8 vs Control 20.69±2.14).

**Conclusion:**

The moderate consumption of non-alcoholic beer will favour night-time rest, due in particular to its hop components, in addition to its other confirmed benefits for the organism.

## Introduction

The hop (*Humulus lupulus* L.), a plant used for brewing because of its aromatic characteristics, has also traditionally been used as a soothing agent. Its sedative activity lies mainly in its bitter acids, and in particular in their oxidative degradation products such as that resulting from the α-acid content: 2-methyl-3-buten-2-ol [1]. Other active components such as the flavonoid xanthohumol are added to degradation products such as 2-methyl-3-buten-2-ol [2], and the terpene, myrcenol [3]. The main mechanism of action of hops is to increase the activity of the neurotransmitter γ-aminobutyric acid (GABA) through modulation of brain GABA(A) receptors [1]. The sedative effect of hops on the nervous system has been widely reported in research using animal models, as also has the narcotic effect at high concentrations due to the 2-methyl-3-[1], [3], [4], [5] buten-ol component [6], [7].

Basic research on hops has found effective applications in the healthy human population as an aid to sleep [8], [9]. In addition to its use in people with sleep problems, the sedative action associated with the components of hops has been used to correct temporary sleep onset and sleep interruption disorders in human populations with treatments applying a combination of valerian and hops [10], [11]. Clinical trials with hops gave satisfactory results as the improvement of sleep quality in patients with insomnia is concerned [12], and in patients suffering from non-organic sleep disorder [13]. Above all, it is also known that both hops and other derivatives of beer can have impact on the inhibitory neurotransmitter GABA(A) [1].

In addition to the central nervous effects of hops as far as GABAergic neurotransmission is concerned, hops does also affect serotonin (5-HT), a further neurotransmitter involved in nocturnal sleep regulation [14]. Moreover, 5-HT is involved as regards the activation of the hormone melatonin, an endocrine agent that entrains circadian rhythms [14], [15], [16]. There are also effects of hops on the neuronal receptors of adenosine which are extensively involved in the mechanism of sleep [17]. Therefore, beer and its hop component are thought to enhance the CNS’s neuroendocrine response via GABA, adenosine, and the biogenic amines serotonin and melatonin with an effective sedative action that both modulates the sleep/wake rhythm and favours the induction of sleep.

In general, there is an initial rejection of the notion that beer may be linked to health because it is a drink that is presumed to cause overweight. However, the caloric content of normal beer is 45 kcal per 100 ml. The caloric content of non-alcoholic beer, which was used in the present study, was relatively low with 17 kcal per 100 ml. Research indicated that moderate daily ingestion of a one-third of a litre tin of beer in women and two tins in men did not produce any significant change in weight [18].

In fact, some properties of beer are thought to have a positive impact on human health. These are thought to be due to the effects of certain components, as for example the aforementioned flavonoid xanthohumol. This compound in particular is thought to fight and prevent cancer as it inhibits the metabolic activation of procarcinogens, induces the activation of anti-cancerigenous enzymes, and inhibits tumour growth in early stages [19], [20]. Other actions of this particular flavonoid include its effective anti-inflammatory effect in inhibiting prostaglandin synthesis via the cyclooxygenases COX-1 and COX-2. Moreover its suppression of the expression of nitric oxide synthase (NOS) whose prolonged activation can trigger the production of vascular endothelial growth factor (VEGF) has been reported [21]. A further property is its antioxidant activity since in vitro xanthohumol has been found more effective than α-tocopherol. Finally a decrease in the tissue damage risk marker, the amino acid homocysteine (HCY), was found. As regards the effects of moderate beer intake major anti-arteriosclerotic, anti-inflammatory, and anti-thrombotic effects have been reported [22].

A further component of beer is 8-prenylnaringenin, a phyto-œstrogen that acts beneficially on bone metabolism, increasing bone density in adults (both men and postmenopausal women), and is thus helpful in preventing or mitigating osteoporosis [23], [24], [25], [26]. Beer can also act as an immunomodulator in healthy populations an increase in leukocytes and T-lymphocyte subpopulations, with this effect being stronger in females than in males), and beer was also shown to be involved in the production of certain cytokines (IL-2,-4,-6,-10; IFN-γ; and TNF-α) and antibodies [27], [28]. The β-bitter acids of the hops, together with myrcenol and xanthohumol, give beer its sedative effect, with their capacity to entrain circadian rhythms, favouring the induction of sleep.

Overall, there are components in beer that are thought to be beneficial to health, and some authors consider that some properties of beer could make it a candidate for possible use as a nutraceutical compound.

Given this context, the main objective of the present work was to study how the hops content of alcohol-free beer – since alcohol is prejudicial to the quality of sleep [29], [30] – may have a sedative action and affect the activity/sleep rhythm in a population subject working rotating and/or night shifts.

## Materials and Methods

### Subjects

The trial was conducted with a sample of 17 female volunteers on the nursing staff of the Extremadura Health Service (*Servicio Extremeño de Salud*, *SES*) who had at least one night shift per working week, and were subject to a high load of work and/or mood stress (as evaluated by means of a questionnaire on job-related stress; Macias et al. 2003). The volunteers were in good health, had normal body weight, and did not take any medications that might have influenced or masked the results of the study. Table 1 gives the anthropometric characteristics of the sample. The study was reviewed and approved by the Ethics Committee of the University of Extremadura, and by the Ethics Committee of the Extremadura Health Service (*SES*).

**Table 1:** Characteristics (X ± SE) of the study sample, (n = 17).

Age (yr)	Weight (kg)	Height (m)	BMI (kg/m^2^)
40.9±2.56	62.4±2.80	1.61±0.10	24.4±1.29

### Experimental Design

The experimental design chosen was one of longitudinal intervention in which each subject was her own control.

The trial period was 2 weeks of “Treatment”, in which the subject ingested 330 mL of alcohol-free beer (*San Miguel 0,0% alcohol*®) with supper.

Prior to starting the trial, the subjects had completed a questionnaire on job-related stress [31]. During the control week and at the end of the treatment with alcohol-free beer, they completed the STAI self-evaluation state-trait anxiety inventory (see below; Spielberger et al. 2008). Throughout the study period, the subjects wore an actimeter (*Actiwatch*®) on the non-dominant wrist. This recorded their daily activity, and allowed us to determine the sleep parameters needed as inputs to the Sleep Analysis® v.5.4 software.

### Parameters Studied

#### Work stress

Once subjects had given informed consent to participate in the study they were asked to complete the work stress questionnaire, as adapted and validated for the Spanish population by Macias et al., [32].

Since participants in the study had to be working under conditions of high stress, we made a quantitative evaluation of the effect of job stress on health in a representative sample of workers of the SES using the Effort Reward Imbalance (ERI) model of Siegrist validated for the Spanish population –“Desequilibrio Esfuerzo-Recompensa” (DER) by Macias et al., [32]. The questionnaire is self-administered and anonymous to ensure confidentiality. The time taken to complete the questionnaire was around 10 minutes.

The following variables were evaluated:

- Effort: The range of scores was from 5 to 25 points, with higher scores being indicative of greater effort experienced by the worker.
- Reward: The range of scores was from 11 to 55 points, with higher scores corresponding to greater job satisfaction.
- Level of Stress: This is the result of dividing extrinsic effort by reward. Participants with values >0.7 were considered to be “stressed”, and with values greater than unity “highly stressed”.
- %Stress: Corresponds to the percentage of the study population presenting stress at the beginning of the study.
- Involvement: A measure of how involved the worker feels in her job. The range of scores was from 6 to 24 points, with a higher the score being indicative for a greater the involvement.

#### State/trait anxiety

Anxiety was quantified using the “State-Trait Anxiety Inventory (Self Evaluation Questionnaire)” (Spielberger et al. 2008). This comprises separate self-assessment scales measuring the two independent concepts of anxiety (state and trait). The time taken to complete the questionnaire was around 10 minutes.

- State Anxiety (S/A): This refers to anxiety “at that specific time.” Conceptualized as a transitory, subjective, emotional state or condition of the human organism, of tension and apprehension, and hyperactivity of the autonomic nervous system.
- Trait Anxiety (T/A): This property is thought to reflect general” Anxiety. Conceptualized as a relative propensity to anxiety in which individuals differ in their tendency to perceive situations as threatening and consequently raise their state anxiety (S/A).

#### Sleep

Sleep related properties were assessed by means of the actimeters (*Actiwatch*®) that the subjects wore on the non-dominant wrist throughout participation, and statistical techniques of time series [33], [34]. analysis (Sleep Analysis® software), we studied the following sleep parameters:

- Time in bed: The time spent in bed.
- Assumed sleep: The difference between the end and the onset of sleep.
- Actual (Real) sleep time: Determined by algorithms, being equivalent to the assumed sleep time minus the time awake.
- Sleep latency: Time before the onset of sleep.
- Sleep-efficiency: The percentage of time asleep while the subject is in bed.
- Total activity: Total number of activity pulses during night-time sleep.

Similarly, using the same actimeter, a non-parametric chronobiological analysis was made of the data [35], applicable to both sinusoidal and non-sinusoidal time series. With this type of analysis, we calculated:

- Interday stability: A measure of the variability of the circadian pattern over the study period. The closer the value to unity, the more robust the rhythm.
- Intraday variability: A measure of the fragmentation of the circadian rhythm; ≤2.
- Relative amplitude: Difference between the mean of the 10 hours of greatest activity, and the mean of the 5 hours of minimum activity in the 24-hour average pattern.

#### Statistical analysis

The Kolmogorov–Smirnov test was applied for examining normality of the distribution of results. Once confirmed that the data did fulfill a normal distribution, Student́s t-test were used to analyze the results. Each value represents the mean ± *SE* from seventeen different volunteers. The degree of significance was set at *p*<.05. All analyses were performed using GraphPad Prism (version 5.0, 2007; GraphPad Software, Inc; San Diego, CA).

## Results

The results of the analysis of the levels of stress for the selection of this study population are given in Table 2. The mean value of the parameter determining stress (Effort/Reward) was 0.93±0.04. This confirmed that the participants were suffering from “stress” as the value was greater than 0.7, and indeed very close to 1, indicating a high level of job stress. Our population was thus indeed suitable for the objective of the study – to measure the evolution of the quality of sleep after the consumption of alcohol-free beer for 14 nights.

**Table 2:** Measures of work stress as assessed using the “Effort Reward Imbalance” (ERI) model.

Effort (E)	Reward (R)	Stress (E/R)	% Stress	Implication
14.84±0.46	36.34±0.65	0.93±0.04	81.5±3.45	15.4±0.73

Self Evaluation Questionnaire (Macias et al. 2003).

(X ± SE) for the study population (n = 17). Stress (E/R)>0.7; high level of stress (E/R) >1.

**Figure 1:**
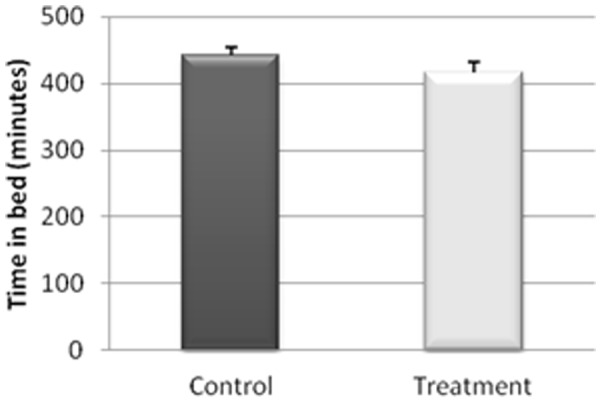
Time in bed during the night-time period of each of the weeks, recorded for 17 work-stressed nurses (X ± S.E.).

**Figure 2:**
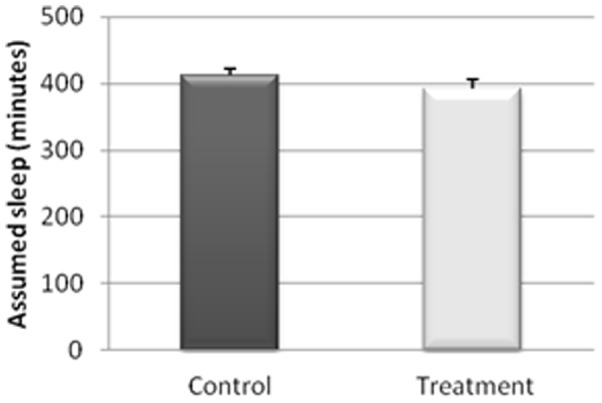
Assumed sleep (including the number of awakenings) of each of the weeks, recorded for 17 work-stressed nurses (X ± S.E.).

**Figure 3:**
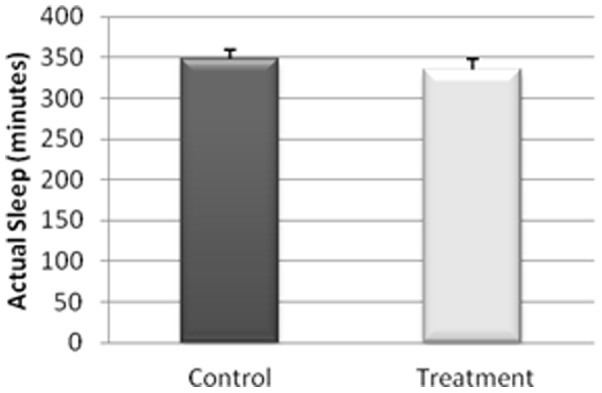
Minutes of real sleep during the night-time period of each of the weeks, recorded for 17 work-stressed nurses (X ± S.E.).

**Figure 4:**
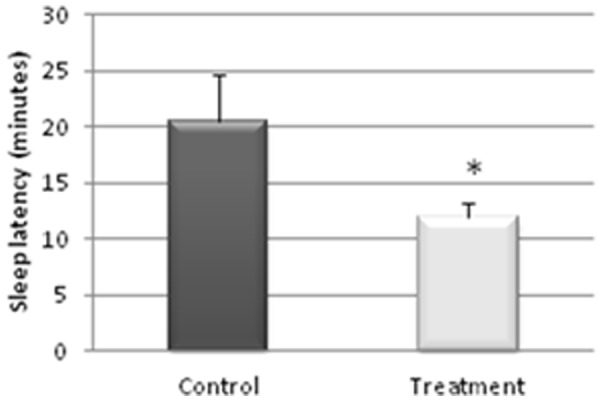
Sleep latency (minutes taken to fall asleep) during the night-time period of each of the weeks, recorded for 17 work-stressed nurses (X ± S.E.). (*) p<0.05 with respect to the values obtained in the Control Week.

**Figure 5:**
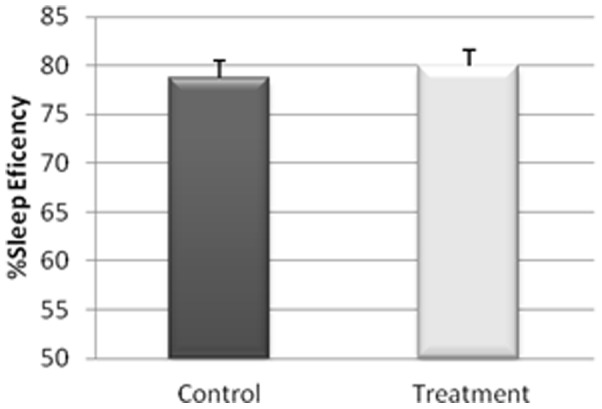
Nocturnal sleep efficiency of each of the weeks, recorded for 17 work-stressed nurses (X ± S.E.).

**Figure 6:**
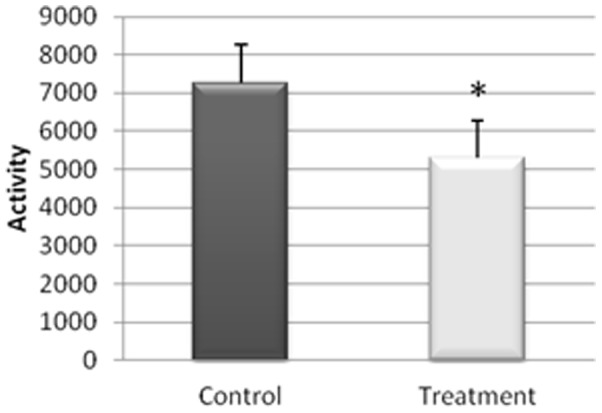
Total activity during the night-time period of each of the weeks, recorded for 17 work-stressed nurses (X ± S.E.). (*) p<0.05 with respect to the values obtained in the Control Week.

**Figure 7:**
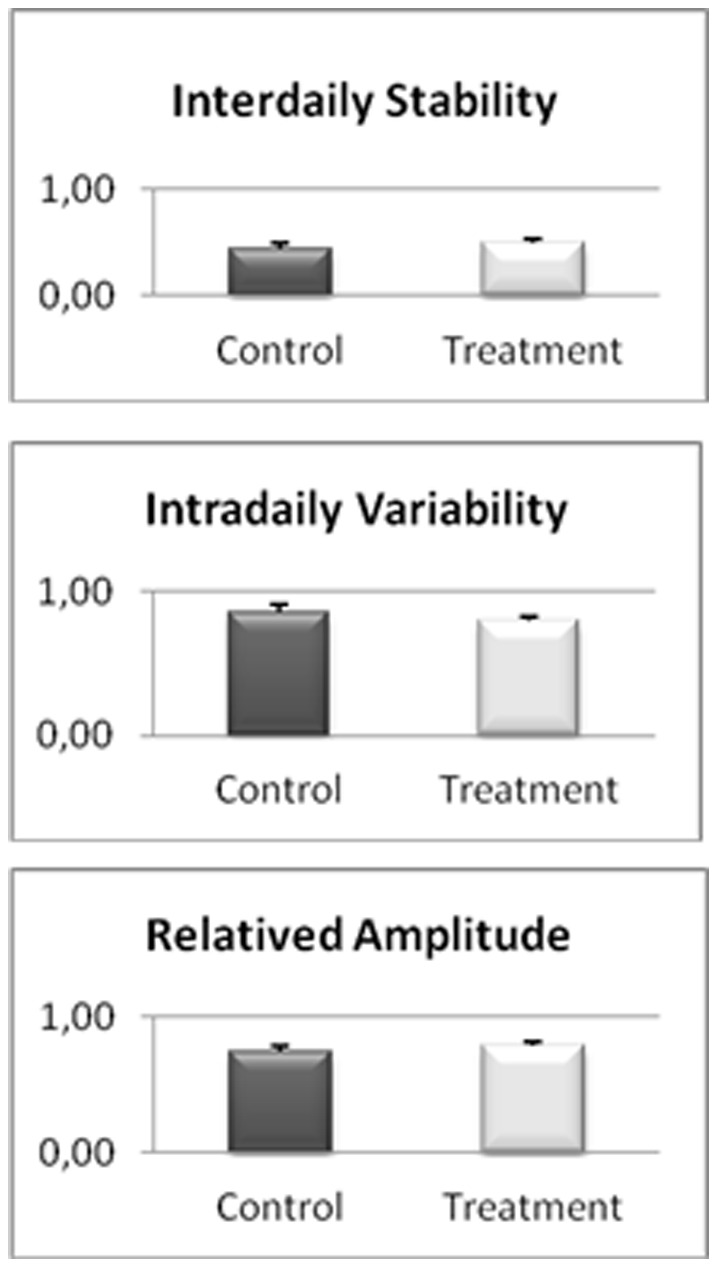
Non-parametric chronobiological parameter; interday stability, intraday variability, and relative amplitude of each of the weeks recorded for 17 work-stressed nurses (X ± S.E.).

The actigraphy results for the study of the sleep/wakefulness rhythm are shown in Figures 1, 2, 3, 4, 5. They indicated that the quality of sleep improved relative to the control group during the two weeks of work stress with ingestion of alcohol-free beer. Although there was no difference in the amount of night-time sleep, the quality of sleep improved as shown by improvement in parameters of night-time sleep. In particular, sleep latency (time taken for the onset of sleep; Figure 4) diminished (p≤0.05) under treatment (12.01±1.19 min) with respect to the control condition (20.50±4.21 min), as also did total activity (p≤0.05; Figure 6; Treatment = 5284.78±836.99 activity pulses vs Control = 7258.78±898.89 activity pulses). In addition in Figure 7, the chronobiological analysis showed increased interday stability (0.51±0.03) in the treatment relative to the control condition (0.45±0.03). Following this ingestion of alcohol-free beer increased the quality of night-time sleep.

With respect to the change of the level of anxiety quantified by means of the STAI (Table 3), we observed that state anxiety (S/A) decreased following under treatment (18.09±3.8) compared to the control period (20.61±2.14).

**Table 3:** Anxiety measures using the “State-Trait Anxiety Inventory” (STAI).

State Anxiety (S/A)		Trait Anxiety (T/A)	
Control	Beer Treatment	Control	Beer Treatment
20.69±2.14	18.09±3.8	19.25±1.68	19.66±1.68

Self Evaluation Questionnaire (Spielberger et al. 2008).

(X ± SE) for the study population (n = 17). The 50th percentile for state anxiety: 21. The 50th percentile for trait anxiety: 21.

## Discussion

The effects of hops on sleep modulation were confirmed in both experimental animal models and human clinical trials. A recent review is given by Zanoli & Zavatti [1]. But to what extent do the levels of the hops present in normal beer have a sedative effect on the organism?

In normal beer the concentration of hops is about 0.3% [36]. Therefore a moderate intake of beer of two 1/3-litre portions per day (666 ml), an amount recommended by several medical scientific societies [18], [27], [37], would contribute about 2 g of hops per day, or 25.7 mg/kg in an average weight human.

In the 1970s, Bravo et al., [38] showed that there was a significant decrease in motor activity in mice after intraperitoneal administration of hop extract, although at rather high doses. Subsequently, its analgesic effect and decrease in spontaneous motor activity were confirmed also in a mouse model, with there being an enhancement in the induction of sleep by pentobarbital [4].

The sedative property of hops has been confirmed in humans, being greater when acting in combination with valerian (*Valeriana officinalis*), with the two acting synergistically on sedative function [3], [8], [9], [10], [39], [40]. Schellenberg et al., [17] studied the combined effects of hops and valerian on central nervous adenosine mechanism, observing an increase in alpha waves while assessing EEG data, with sleep inducers being generated through the adenosine receptors. To this action on adenosine, one must also add the hypnotic effect on the CNS through receptors for serotonin [16] and the hormone melatonin [14], [15], [41], which are involved in sleep and circadian rhythms, respectively.

Our study in a population of health professionals experienceing a considerable amount of work stress showed that, even though there was no increase in the length of repose in bed, there was a clear improvement in the quality of night-time sleep after the ingestion of beer at the end of the day. This is illustrated by the reduction in sleep latency and the notable reduction in nocturnal mobility, thereby achieving restful sleep which was reflected in decreased anxiety [42].

We would note that a greater hop content of the beer, but without increasing the alcohol content, could be expected to have led to a greater sedative action, both earlier and faster, in our population, but always at moderate doses since otherwise there could arise unwanted effects. The reason for this opinion is that other work, applying substantially higher *Humulus lupulus* concentrations (800 mg/kg = 160 mg dose), and in association with anæsthetics and hypnotics such as ketamine, led to prolonged states of deep narcosis [43]. Similarly, the product of the oxidative degradation of the α-acid content of fresh hops, 2-methyl-3-buten-2-ol, applied to mice at concentrations of 0.8 g/kg produced narcosis that lasted 8 hours [7]. In other study by Zanoli et al. [44], oral administration of hop extracts to mice achieved a reduction in spontaneous locomotor activity. In particular, extract concentrations of 10 and 20 mg/kg b.w. were used, and increased sleep time and reduced motility compared to control animals were observed.

In a biphasic animal model, the common quail (with, like humans, a nocturnal period of sleep and diurnal activity), we have studied the effect of hop extract concentrations similar to those in beer, observing a decrease in motor activity during the night [45].

The results thus suggest that, because of its hop content, beer may have a possible use as a sedative in humans. The mechanism would principally be by modulating the GABAergic response through the effects of the hop components myrcenol [3], xanthohumol, and such α-acid derivates [1] as 2-methyl-3-buten-2-ol.

As with other food components, beer should always be only moderately consumed. Its use in this way and at supper-time could and should represent a nutritional tool in the discipline of chrononutrition, since its neuromodulatory components help entrain the circadian sleep/wake rhythms [34]. Thus future lines of research will be to examine the possibility of achieving a higher level of sedation and reducing anxiety with the ingestion at supper-time of alcohol-free or low alcohol content beer with a higher hop content than we tested in the present work.

Through its hop content, alcohol-free beer could exert a sedative action in humans, apart from its benefits for health when consumed in moderation [24], among which are its anti-cancerigenous [46] and cardiovascular health [23], [47], [48] properties. One can therefore conclude that a moderate consumption of non-alcoholic beer will favour night-time rest, due in particular to its hop components, in addition to its other confirmed benefits for the organism.
